# Microbial Community Structure in Contrasting Hawaiian Coastal Sediments

**DOI:** 10.1007/s00248-025-02548-7

**Published:** 2025-05-23

**Authors:** Benjamin Van Heurck, Diana Vasquez Cardenas, Astrid Hylén, Emilia Jankowska, Devon B. Cole, Francesc Montserrat, Matthias Kreuzburg, Stephen J. Romaniello, Filip J. R. Meysman

**Affiliations:** 1 Geobiology Research Group, Department of Biology, University of Antwerp, Universiteitsplein 1, 2610 Antwerp, Belgium; 2 Vesta, PBC, San Francisco, CA USA; 3 Present Address: Hourglass Climate, NPO, Montclair, NJ USA; 4 Present Address: Department of Marine Chemistry, Trace Gas Biogeochemistry, Leibniz-Institute for Baltic Sea Research, Warnemünde, Germany; 5 Present Address: ARK Rewilding Nederland, Winselingseweg 95, 6541 AH Nijmegen, The Netherlands

**Keywords:** Microbial communities, Sediment geochemistry, Coastal sediments, 16S rRNA amplicon sequencing, Olivine, Coastal enhanced weathering

## Abstract

Microbe-mineral interactions play a fundamental role in marine sediments and global biogeochemical cycles. Here, we investigated the sediment microbial communities in two contrasting field sites on Big Island, Hawaii (USA), that differ in their bay morphology and sediment grain size distributions: Papakōlea Beach (exposed, finer sediment) and Richardson Ocean Park (sheltered, coarser sediment). We selected three stations within each bay and characterized the mineral and chemical composition of the sediment and porewater, and used 16S rRNA amplicon sequencing of the V4V5 hypervariable region to investigate the naturally occurring microbial communities. Microbial community structure differed significantly between the two bays, rather than within each bay, whereby microbial diversity was markedly lower at Papakōlea compared to Richardson. We correlated environmental variables to microbial community structure in order to identify the key drivers of community differences between and within the two bays. Our study suggests that differing physico-chemical properties of the sediment and porewater, resulting from the contrasting bay morphologies and geophysical drivers, are the main factors influencing microbial community structure in these two bays. Papakōlea Beach is a naturally occurring “green sand” beach, due to its high olivine content. This site was selected in the broader context of a field campaign investigating olivine as a source mineral for ocean alkalinity enhancement (OAE), a carbon dioxide removal technology. Our results highlight the complexity of marine sediment environments, with implications for the monitoring, reporting and verification of future field trials involving olivine addition for ocean alkalinity enhancement.

**Supplementary Information:**

The online version contains supplementary material available at 10.1007/s00248-025-02548-7.

## Introduction

Big Island (Hawaii, USA) consists of five distinct volcanoes [1]. Therefore, the island contains large quantities of basalt rock and olivine, which physically weather and contribute to beach sediments. Likewise, calcifying organisms (e.g., corals and calcareous algae) are abundant in the coastal waters surrounding Big Island, thus contributing to the formation of carbonate sand [2]. Consequently, beaches on the island vary substantially in their mineralogical composition and consist either predominantly of silicate sand, carbonate sand, or a mix of the two.

Microbe-mineral interactions are fundamental to marine sediments and play an important role in global biogeochemical cycling [3, 4]. Microorganisms alter the physical structure, geochemistry and stability of the sediment matrix through degradation of organic matter, biofilm formation, release of cellular exudates, and the precipitation and dissolution of minerals [5–7]. Conversely, differences in sediment properties such as grain size also shape microbial communities by determining the permeability of the sediment, and thus the availability of metabolic substrates (e.g., organic carbon and oxygen) through porewater irrigation [4, 8, 9].

Permeable, sandy sediments are formed in areas with strong hydrodynamic disturbance and are characterized by strong advective porewater flushing and high bed shear stress [10, 11]. This results in deep oxygen penetration which fosters the development of a uniform microbial community with sediment depth, and the prevalence of aerobic metabolisms [12–15]. Increased bed shear stress additionally prevents the formation of benthic biofilms [16]. Consequently, coastal permeable sediments with similar grain size, organic carbon content, and wave exposure tend to display similar microbial communities in the top centimeters [17].

The chemical composition of minerals and differences in their grain surface roughness can also impact the microbial community structure. Silicate minerals typically have a lower affinity for bacterial colonization compared to carbonate substrates, primarily due to their negatively charged surfaces that repel the negatively charged cell walls of bacteria [18]. Grain topography is additionally important, whereby rougher grains (more indents and grooves) are more densely populated and display more diverse communities compared to smooth, convex grains [9]. Co-occurrence of different sand types may therefore cause high levels of community heterogeneity and increase the availability of microbial niches [19].

Here, we study the microbial community in two separate bays on Big Island, Hawaii: Papakōlea Beach is a naturally occurring olivine “green sand” beach located in the southernmost part of Big Island and is exposed to the open Pacific Ocean, consequently experiencing strong hydrodynamic disturbance. Richardson Ocean Park is a contrasting field site, located on the eastern side of Big Island, in a bay sheltered from high hydrodynamic disturbance by lava rock beds that present sizeable coral reefs. As a result, the sand consists of basalt, olivine, and carbonates, but with an expected lower olivine content compared to Papakōlea. We compare microbial communities in these two contrasting bays and assess the impact of sediment mineralogy, grain size and porewater geochemistry on microbial community structure, providing insight into the environmental factors that drive microbial diversity in permeable coastal sediments.

This study is part of a broader field campaign investigating Papakōlea Beach as a natural analogue for ocean alkalinity enhancement (OAE) via coastal enhanced weathering (CEW) of olivine. This technique aims to remove atmospheric carbon dioxide (CO_2_) through the deposition of finely pulverized silicate minerals, such as olivine, in coastal environments. Chemical weathering of these minerals generates alkalinity, which increases the CO_2_ storage capacity of the coastal ocean [20, 21]. Olivine content at Papakōlea can reach up to 70% in some areas [22]. Although our study does not focus exclusively on olivine, Papakōlea Beach provides a unique opportunity to investigate microbial communities in a naturally occurring olivine-rich environment, which is otherwise rare in coastal settings because of olivine’s high weathering rate.

## Methods

### Sample Collection, Physical and Geochemical Characterization

**Fig. 1:**
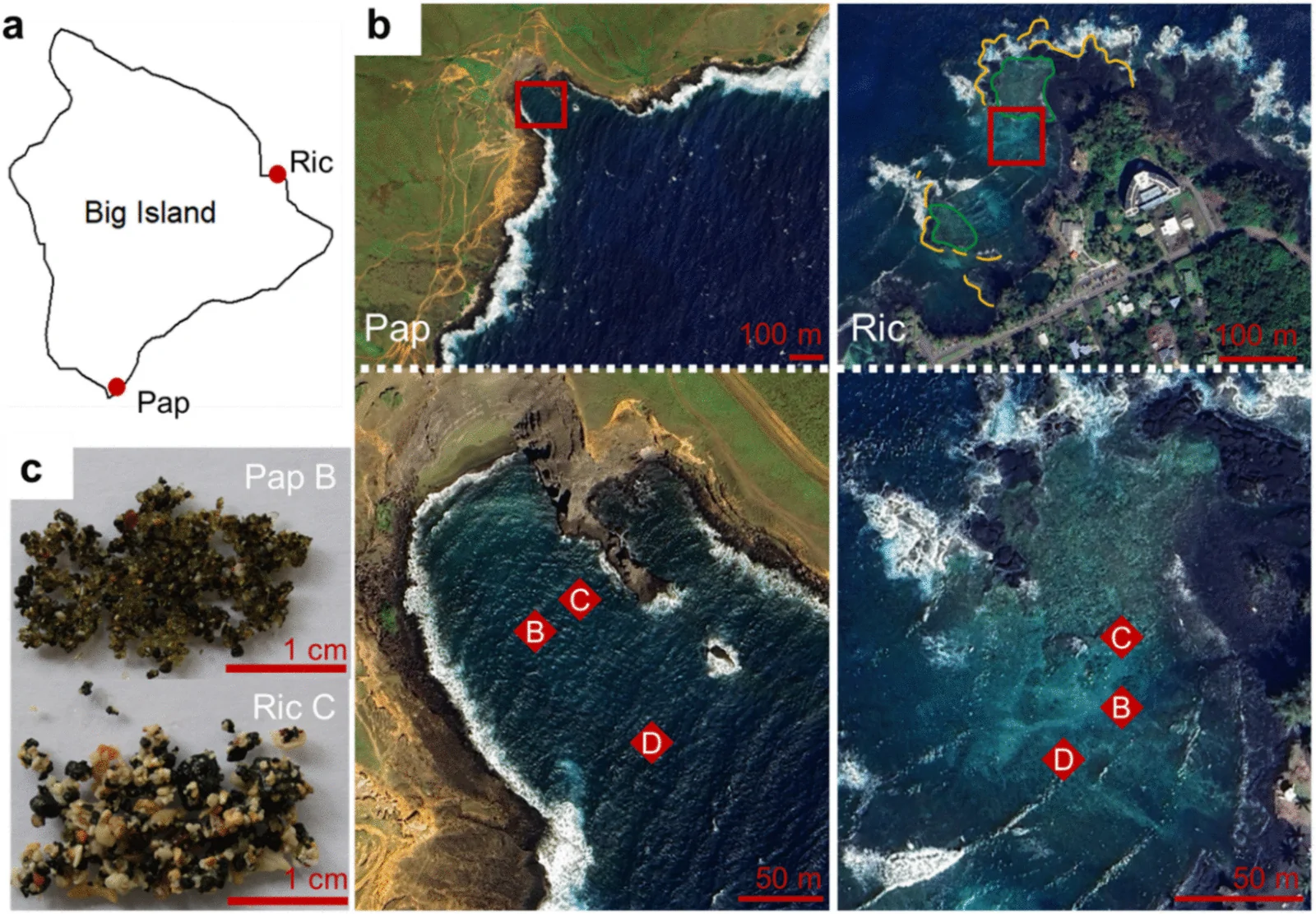
**a** Outline of Big Island, Hawaii (USA) with indication of field sites: Papakōlea (Pap) and Richardson (Ric). **b** Overview of each bay. In the top panels, the red square indicates the location of the sampled stations. At Richardson, yellow lines indicate rock beds sheltering the bay and green areas indicate coral reefs. Bottom panels show a close up of each bay; red diamonds indicate the three stations where sediment was sampled (B, C and D). **c** Pictures of representative sediment from stations Pap B and Ric C. The outline map was made with the R *maps* package (version 3.4.2); map images were modified from Google Earth pro (version 7.3.6.9796)

Three stations were selected randomly in each bay, where water depth, salinity, and temperature were measured, and a visual description of the sediment was recorded (Fig. 1a–c; Table 1). At Papakōlea, cores were collected from the middle of the bay (Pap B, Pap C), and in the opening of the bay (Pap D). At Richardson, cores were collected from the northern part of the embayment (Ric B, Ric C, and Ric D), whereby station C was located closest to the coral reef.

**Table 1:** Water depth, sediment description, temperature, salinity, and location of all sampled stations

Site	Station	Water depth (m)	Sediment description	Temperature (°C)	Salinity	Latitude, longitude (°)
Papakōlea (Pap)	B	5.8	Silicate dominant (mostly green sand) Coarse(r) grains	26	33	18° 56′ 07.40″, − 155° 38′ 45.15″
	C	5.2	Silicate/carbonate mix Fine grains	27	33	18° 56′ 07.82″, − 155° 38′ 44.33″
	D	7.4	Silicate/carbonate mix Fine grains	27	34	18° 56′ 05.17″, − 155° 38′ 42.84″
Richardson (Ric)	B	3.0	Silicate/carbonate mix Coarse grains	27	33	19° 44′ 11.5103″, − 155° 00′ 50.07″
	C	2.8	Carbonate dominant (mostly white sand) Coarse grains	26	34	19° 44′ 12.34″, − 155° 00′ 50.07″
	D	3.0	Silicate/carbonate mix Coarse grains	28	33	19° 44′ 10.86″, − 155° 00′ 50.85″

In July and August 2022, sediment cores (inner diameter 4 cm) were collected manually by SCUBA divers at each station. Sediment cores were always collected in the troughs of sediment ripples. For chlorophyll-*a* analysis, three separate cores were collected per station, and the first 5 cm were placed in aluminum foil-covered plastic bags to prevent exposure to sunlight, before storage at − 20 °C. Chlorophyll-*a* content was determined by fluorescence (EPA Method 445.0) [23] at the University of Hawai’i, Hilo (Hawaii, USA) [24]. A fourth core was taken to sample a deep sediment layer (9–15 cm at Pap B, 15–21 cm at Pap C-D, and 9–12 cm at all Richardson stations) for physical characterization of the sediment. The deeper sediment layers were used to evaluate the grain size distribution by sequential sieving of wet sediment (wt.%; < 63 µm, 63 µm – 1 mm and > 1 mm), and the mineralogical composition (wt.%) determined by X-ray diffraction (XRD) (QMineral, Leuven, Belgium).

Triplicate porewater samples were collected by SCUBA divers using carbon fiber sippers (MHE products, East Tawas, MI, USA) attached to plastic syringes sealed with a Luer stopcock. Carbon fiber sippers were inserted at 2, 5, 10, 15, 20, and 25 cm depth (Fig. S1) and moved 1 m between replicate porewater profiles. Dissolved oxygen was measured immediately in the field using a PyroScience FireSting®-GO2 meter (PyroScience GmbH, Aachen, Germany) and probe calibrated using ambient air and an Oakton Zero Oxygen Calibration standard (Environmental Express, Charleston, SC, USA). The remaining porewater was filtered through a 0.45 µm Supor™ filter and preserved by freezing at − 20 °C for nutrient analysis. Nutrient samples were analyzed within 1 week at the University of Hawai’i, Hilo Analytical Laboratory using a Lachat Quikchem 8500 Series II Flow Injection Analyzer (Hach Co., Loveland, CO, USA) optimized for seawater nutrient analysis.

For microbial analysis, a fifth sediment core per station was sectioned at 2 cm resolution for the first 6 cm and at 3 cm resolution for the remaining depth (max. 21 cm, Table S1). All equipment was sterilized with 70% ethanol between slices. Sediment slices were homogenized and triplicate subsamples of ~ 1 mL sediment were collected in sterile 2 mL Eppendorf® tubes. At Ric D, only one sample was collected per sediment slice. Samples were kept in the dark and cooled in the field, frozen at − 20 °C within 6 h, shipped on dry ice (1 month after collection) and stored at − 80 °C until further processing.

### DNA Extraction and Amplicon Sequencing

DNA was isolated from one replicate sediment sample (~ 0.5 g) per depth layer using the DNeasy® PowerSoil® Pro Kit (Qiagen, Hilden, Germany). The V4 V5 hypervariable region of the 16S rRNA gene was PCR amplified using universal bacterial primers 515 F-Y (5′-GTGYCAGCMGCCGCGGTAA) and 926R (5′-CCGYCAATTYMTTTRAGTTT) [24]. PCR was run on a Bio-Rad T100™ Thermal Cycler (Bio-Rad Laboratories, Hercules, CA, USA). Samples were sequenced using an Illumina MiSeq sequencer (Eurofins Genomics, Konstantinz, Germany), generating 2 × 300 bp paired-end reads. Raw sequencing data were uploaded to the NCBI SRA Bioproject PRJNA1217334. More details on microbial sample processing are provided in the supplementary methods (Online Resource).

### Microbial Community Analysis

The DADA2 pipeline (version 1.26.0; [25]) was implemented to process the obtained Illumina sequence reads (mean sequencing depth 78,818 ± 19,527 reads; Table S1), resulting in an amplicon sequencing variant (ASV) table. Taxonomy was assigned against the SILVA reference database (version 138; [26]) using the built-in naïve Bayesian classifier method [27]. Chloroplast sequences in the microbial dataset, with a relative abundance greater than 0.1%, were identified using BLASTn. The diversity of microbial communities was quantified using the Shannon diversity index (R *microbiome* package version 1.20.0; [28]) and the difference in diversity between sites was assessed using a Wilcoxon rank sum test. Community similarity was analyzed with non-metric multidimensional scaling (NMDS) using Bray–Curtis dissimilarity (R *phyloseq* package, version 1.42.0; [29]). Environmental variables were fitted to the NMDS using “envfit” (R *vegan* package; version 2.6–4; [30]). Prior to running “envfit,” a log(x + 1) transformation was applied to the nutrient and chlorophyll-*a* data and a centered log-ratio (CLR) transformation to the grain size and mineralogy data. Then, porewater data (DO, nutrients) were binned to the corresponding microbial sample depths, and mineralogical, grain size and chlorophyll-*a* data were extrapolated to match all microbial sampling depths (Table S1). Differences in community structure between the two bays and between the surface (0–6 cm) and deeper layers (> 6 cm) within each bay were statistically tested using analysis of similarities (ANOSIM) (R *vegan* package; version 2.6–4; [30]). Functional Annotation of Prokaryotic Taxa (FAPROTAX, version 1.2.7; [31]) was used to identify the putative metabolic functional potential of the microbial communities. FAPROTAX assigns prokaryotic taxonomy to putative metabolic functions based on current literature on cultured strains. It is important to note that taxa can be assigned to multiple putative metabolic functions, and these functions can be nested within each other (e.g., aerobic chemoheterotrophy nested in chemoheterotrophy). More detailed information on data processing is provided in the supplementary methods (Online Resource).

## Results

### Sediment Characterization

**Fig. 2:**
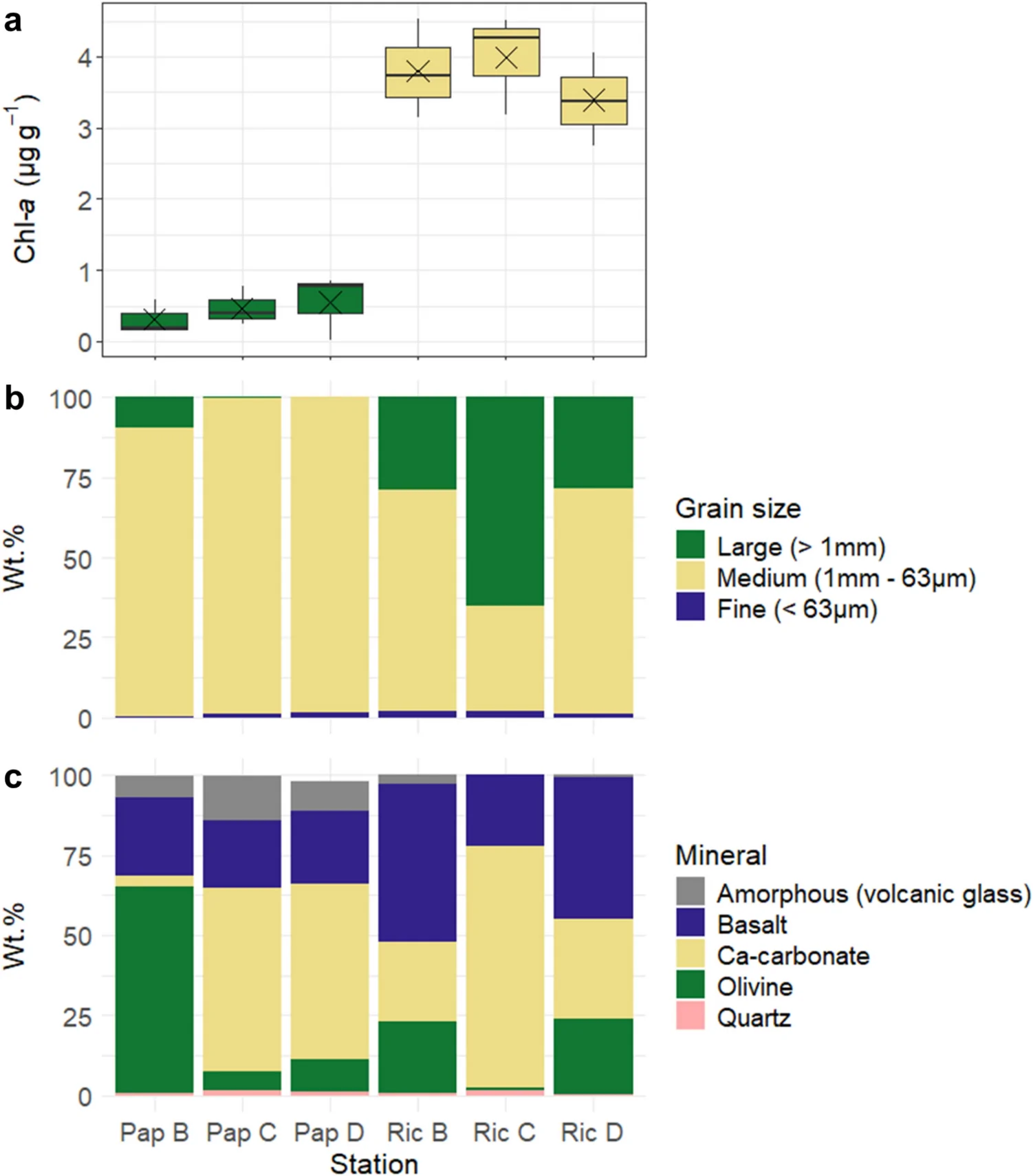
Sediment chlorophyll-*a* content (µg g^−1^) (**a**), grain size distribution (wt.%) (**b**), and mineralogical composition (wt.%) (**c**) of sampled stations in Papakōlea and Richardson bays. Sample labels on the X-axis indicate the site (Pap or Ric) and station (B, C, or D). X in panel a indicates the mean chlorophyll-*a* content (µg g^−1^) per station

Clear differences were observed between the two bays with regard to chlorophyll-*a* content, grain size distribution and mineralogy. Average chlorophyll-*a* content across stations was an order of magnitude lower at Papakōlea than at Richardson (0.4 ± 0.3 versus 3.7 ± 0.7 µg g^−1^, respectively; Fig. 2a, Table S2). Grain size analysis showed a smaller large fraction (> 1 mm) at Papakōlea (0.1–10 wt.%) compared to Richardson (29–65 wt.%; Fig. 2b, Table S2). The mineralogical analysis of sediments from both bays showed a varying mixture of olivine, basalt, and carbonate sands across all stations (Fig. 2c). Noteworthy were the high olivine content of station Pap B (64 wt.%) and the high carbonate content of station Ric C (75 wt.%). Furthermore, stations Pap C and D showed a higher carbonate content (55 and 57 wt.%) compared to stations Ric B and D (25 and 31 wt.%). Conversely, the olivine content at stations Pap C and D (6 and 10 wt.%) was lower than at stations Ric B and D (22 and 23 wt.%). Large calcareous fragments were observed at all Richardson stations but were absent at Papakōlea (Fig. 1c, Fig. S2).

Plagioclase (8–22 wt.%), clinopyroxene (8–20 wt.%), and orthopyroxene (2–7 wt.%) are the silicate mineral components of basalt rock and were therefore combined into one fraction (Fig. 2c, Table S2). Basalt was present in similar proportions at Papakōlea (21–24 wt.%), whereas at Richardson the sediment content of basalt was more variable (22–49 wt.%). Minor silicate mineral components across both bays were amorphous volcanic glass (0–14 wt.%) and quartz (0.5–1 wt.%). At Papakōlea, higher fractions of amorphous volcanic glass (7–14 wt.%) were found, which were mostly absent at Richardson (0–2.9 wt.%). The carbonate fractions consisted of magnesium calcite and aragonite (4–75 wt.% combined; Fig. 2c, Table S2). The presence of halite was a sampling artefact caused by the drying of samples containing residual seawater and was removed from plots (Table S2).

### Chemical Characterization

**Fig. 3:**
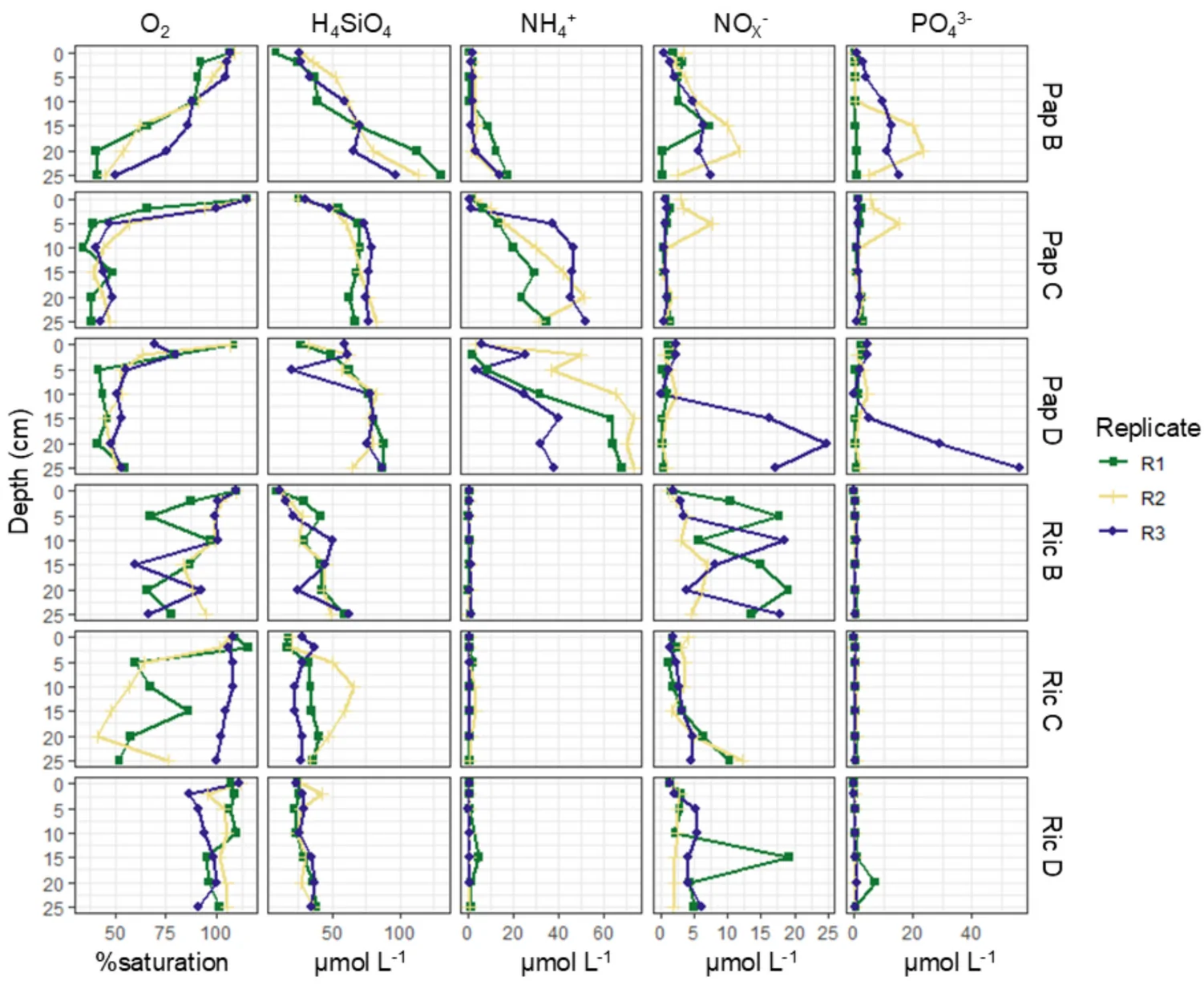
Porewater profiles for oxygen and nutrients at Papakōlea and Richardson. From left to right: oxygen (O_2_), dissolved silica (H_4_SiO_4_), ammonium (NH_4_^+^), nitrate + nitrite (NO_x_^−^), and phosphate (PO_4_^3−^). The Y-axis displays the depth (in cm), the X-axis displays concentration values (% saturation for O_2_, µmol L^−1^ for nutrients), and colors and shapes indicate triplicate depth profiles

In both bays, oxygen was present throughout the sediment, suggesting strong and deep physical irrigation of the permeable deposits. At stations Pap C and D, the oxygen saturation declined within the top 5 cm, then remained constant at ~ 50% (5–25 cm). At Pap B, where the sediment contained a larger coarse fraction, oxygen remained fully saturated within the top 5 cm, declining more gradually, reaching ~ 50% at 20–25 cm depth. At Ric D, the oxygen saturation stayed consistently near 100% across the whole sediment profile. At Ric B, the oxygen saturation varied between replicates and depths but remained within the 50–100% range throughout the sediment. At Ric C, variation was large between replicates, with R1 and R2 declining rapidly in the top 5 cm, then remaining stable at ~ 50% (5–25 cm), whereas in R3, oxygen saturation remained ~ 100% across the whole sediment depth (Fig. 3; Table S3).

Dissolved silica (H_4_SiO_4_) concentrations in the porewater increased with depth at all stations and were double at Papakōlea (max. 88–130 µmol L^−1^) compared to Richardson (max. 43–65 µmol L^−1^) (Fig. 3; Table S3). Ammonium accumulated with depth at Pap C and D, reaching up to ~ 60 µmol L^−1^, though with considerable variation between replicates, particularly at deeper depths. At Pap B, ammonium (NH_4_^+^) remained low within the top 15–20 cm, and then slowly increased to reach ~ 20 µmol L^−1^. At Richardson, the porewater was consistently depleted of ammonium, which did not exceed 5 µmol L^−1^ at any of the stations (Fig. 3; Table S3). The nitrate/nitrite (NO_x_^−^) concentration profiles did not exhibit a clear trend with depth and were comparable between the two bays. At Papakōlea, the maximum concentrations recorded were 8–25 µmol L^−1^, though with noticeable variation between replicates. At Richardson, the maximum concentrations remained within the 12–19 µmol L^−1^ range (Fig. 3; Table S3). Phosphate (PO_4_^3−^) was present across all Papakōlea stations (max. 15–56 µmol L^−1^), though variability between replicates was considerable, especially at Pap D. In contrast, at Richardson, phosphate was depleted (similar to ammonium), not exceeding 1 µmol L^−1^ at Ric B and C and reaching a maximum of 7 µmol L^−1^ at Ric D (Fig. 3; Table S3).

### Microbial Characterization

The DADA2 pipeline resulted in a total of 20,254 unique ASVs after singleton removal, across the whole dataset. Sample Pap C 6–9 cm had the lowest number of ASVs (326) and sample Pap C 0–2 cm had the highest (2568 ASVs; Table S1).

**Fig. 4:**
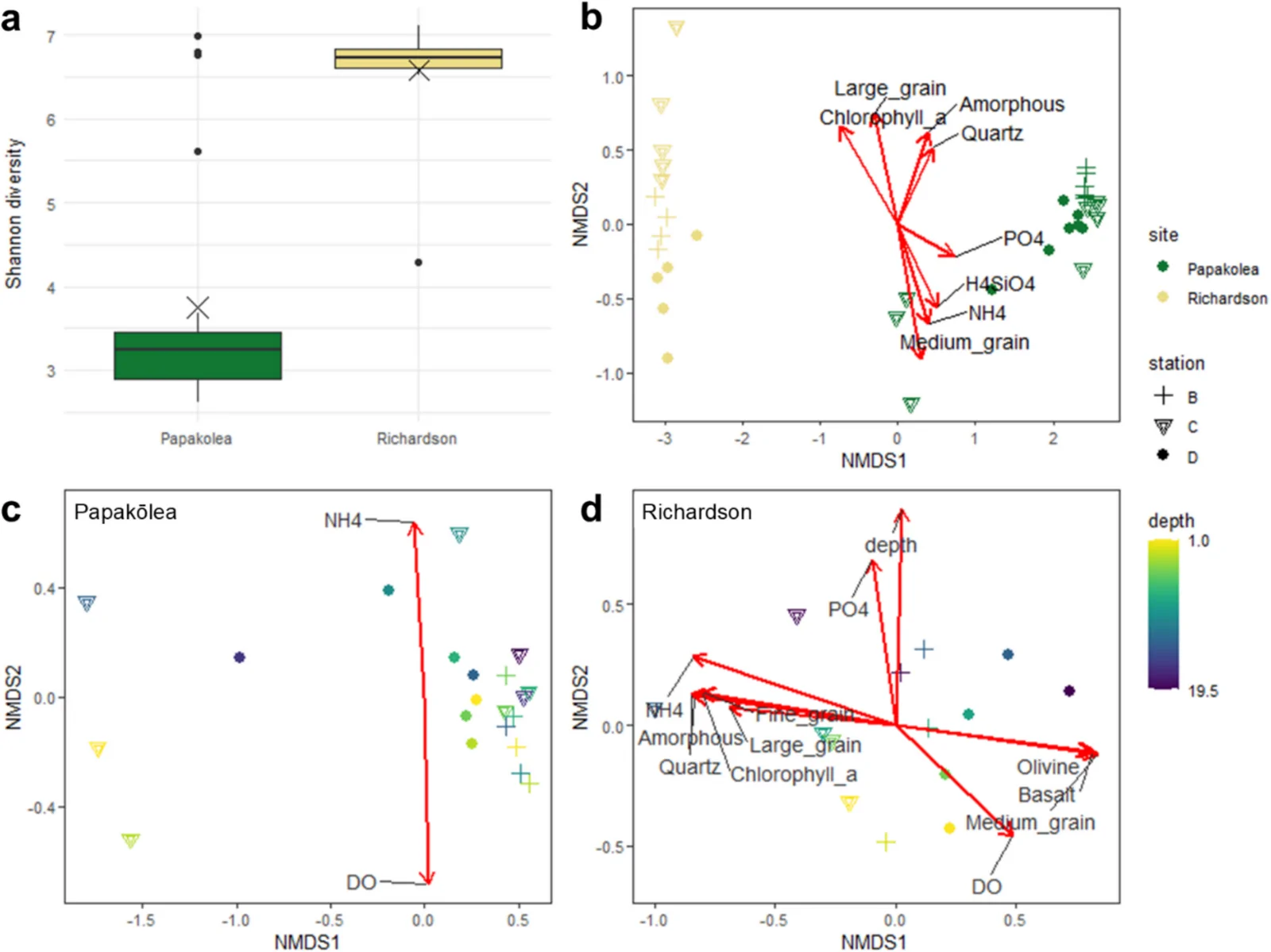
**a** Boxplot displaying Shannon diversity at Papakōlea and Richardson. Closed circles indicate outliers, X indicates the mean Shannon diversity per site. **b**, **c**, **d** NMDS ordination plots of sediment microbial communities, based on Bray–Curtis dissimilarity: **b** Papakōlea and Richardson combined, **c** Papakōlea, and **d** Richardson. Stress values for the NMDS ordinations are 0.04, 0.05, and 0.11, respectively. In **b**, colors indicate sampling sites; in **c** and **d**, colors represent depth. Markers indicate sampling stations. Vectors in the ordination plots indicate significant correlations with environmental variables (*R*^2^ > 0.4, *p* < 0.05), with arrow length representing the strength of the correlation

The microbial communities differed significantly between the two bays, both with regard to their Shannon diversity and taxonomic composition. The Shannon diversity index was half at Papakōlea (3.8 ± 1.4) compared to Richardson (6.6 ± 0.7; *p* < 1e − 07, Fig. 4a; Table S1). ANOSIM analysis further supports the clear separation of microbial community structure between the two bays (*R* = 0.99, *p* < 1e − 04). No significant difference in community structure between the surface (0–6 cm) and deeper (> 6 cm) sediment layers was observed at Papakōlea (*R* = 0.022, *p* = 0.31), thus suggesting a homogenous distribution of microbes in the surface layer (up to 20 cm). In contrast, at Richardson, microbial communities differed moderately between the surface and deeper layer (*R* = 0.37, *p* < 0.004).

NMDS scaling further supports a clear separation of microbial communities based on site, along the first axis, whereas the second axis separates Richardson samples by station, an effect that is not apparent at Papakōlea (Fig. 4b). The incorporation of environmental variables via “envfit” provides additional insight into the factors that correlate with microbial community structure, between and within each site. For Papakōlea and Richardson combined, the highest significant correlations were observed for chlorophyll-*a* (*R*^2^ = 0.96, *p* = 0.001), medium grain (*R*^2^ = 0.90, *p* = 0.001), and large grain (*R*^2^ = 0.65, *p* = 0.001). More moderate correlations were observed for ammonium (*R*^2^ = 0.59, *p* = 0.001), phosphate (*R*^2^ = 0.57, *p* = 0.001) and silica (*R*^2^ = 0.56, *p* = 0.001). These correlations explain the separation of Papakōlea and Richardson along the NMDS1 axis and highlight the observed differences in chlorophyll-*a* content, grain size and nutrient concentrations (Figs. 2a, b and 3) as potential drivers of microbial community structure between the two bays.

At Papakōlea, no clear clustering of samples was observed (Fig. 4c) and only dissolved oxygen (DO) and ammonium showed a moderate correlation (*R*^2^ > 0.4) with the NMDS ordination. Other significant, but weak correlations (*R*^2^ < 0.4) were found for some mineralogical components (olivine, ca-carbonate, quartz, amorphous volcanic glass), and silica (Table S4). In contrast, the NMDS of Richardson (Fig. 4d) further explains the observed separation along the NMDS2 axis of the combined ordination (Fig. 4b). The NMDS1 axis separated samples by station, with strong correlations (*R*^2^ > 0.4) for mineralogical components (olivine, basalt, quartz, amorphous volcanic glass), grain size (fine grain, medium grain), chlorophyll-*a*, DO, and ammonium (Table S4). The NMDS2 axis correlates strongly with depth (*R*^2^ = 0.8, *p* = 0.001), indicating that microbial community structure at Richardson is influenced by spatial variation in sediment mineralogy and grain size, as well as depth.

**Fig. 5:**
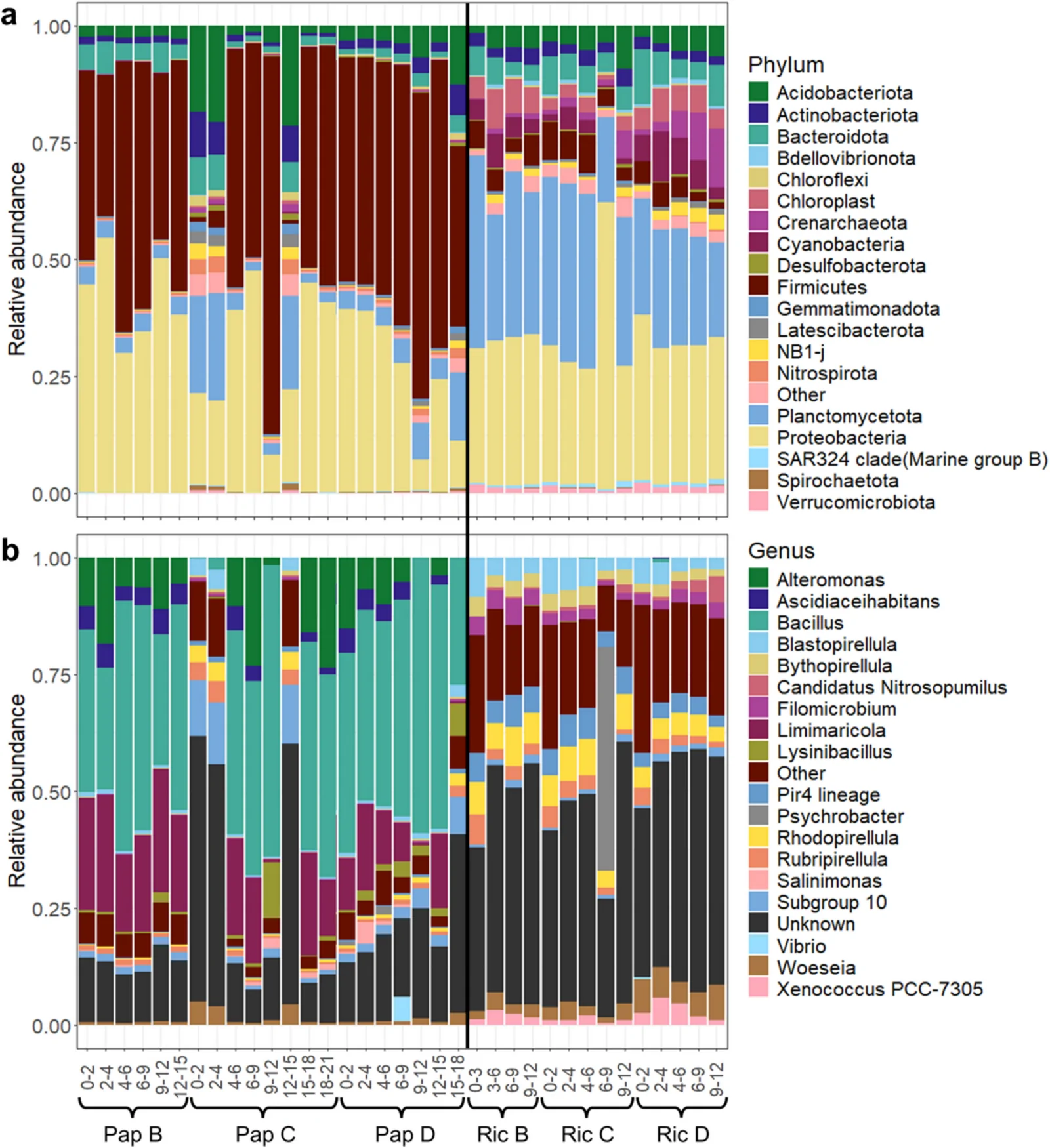
Depth profiles of the 20 most abundant microbial taxa at **a** phylum, and **b** genus level for Papakōlea (Pap) and Richardson (Ric). Sites (Pap or Ric), stations (B, C, or D), and sediment depths in cm (e.g., 0–2, 2–4) are indicated in sample labels. The black line separates the two sites

Although the dominant groups at phylum level were similar between both bays (Acidobacteriota, Cyanobacteria, Bacteroidota, Firmicutes, Planctomycetota, and Proteobacteria; Fig. 5a), clear differences were observed in their relative abundances. Notably, the average relative abundances of Cyanobacteria and Planctomycetota were 10 and 4 times lower at Papakōlea (0.03 ± 0.06% and 7 ± 7%, respectively) than at Richardson (4 ± 3% and 30 ± 7%, respectively). Likewise, the average relative abundance of Firmicutes also differed by one order of magnitude between the two bays (Papakōlea: 44 ± 20%, Richardson: 5 ± 2%). At Papakōlea, four genera (*Alteromonas*, *Ascidiaceihabitans*, *Bacillus*, and *Limimaricola)* made up approximately 80% of the microbial community in most samples, with approximately 50% of the community consisting of *Bacillus* (phylum Firmicutes), primarily corresponding to a single ASV. In contrast, no single genus dominated the community at Richardson, consistent with the higher Shannon diversity observed (Figs. 4a and 5b). Additionally, the relative abundance of eukaryotic chloroplast sequences was markedly lower at Papakōlea (0.1 ± 0.2%) compared to Richardson (5 ± 2%). A BLAST search of chloroplast sequences from Richardson with relative abundance > 0.1% (*N* = 15, representing 70% of Richardson chloroplast sequences), revealed a diverse phototroph community, including foraminifera, diatoms, and brown algae (Table S5).

**Fig. 6:**
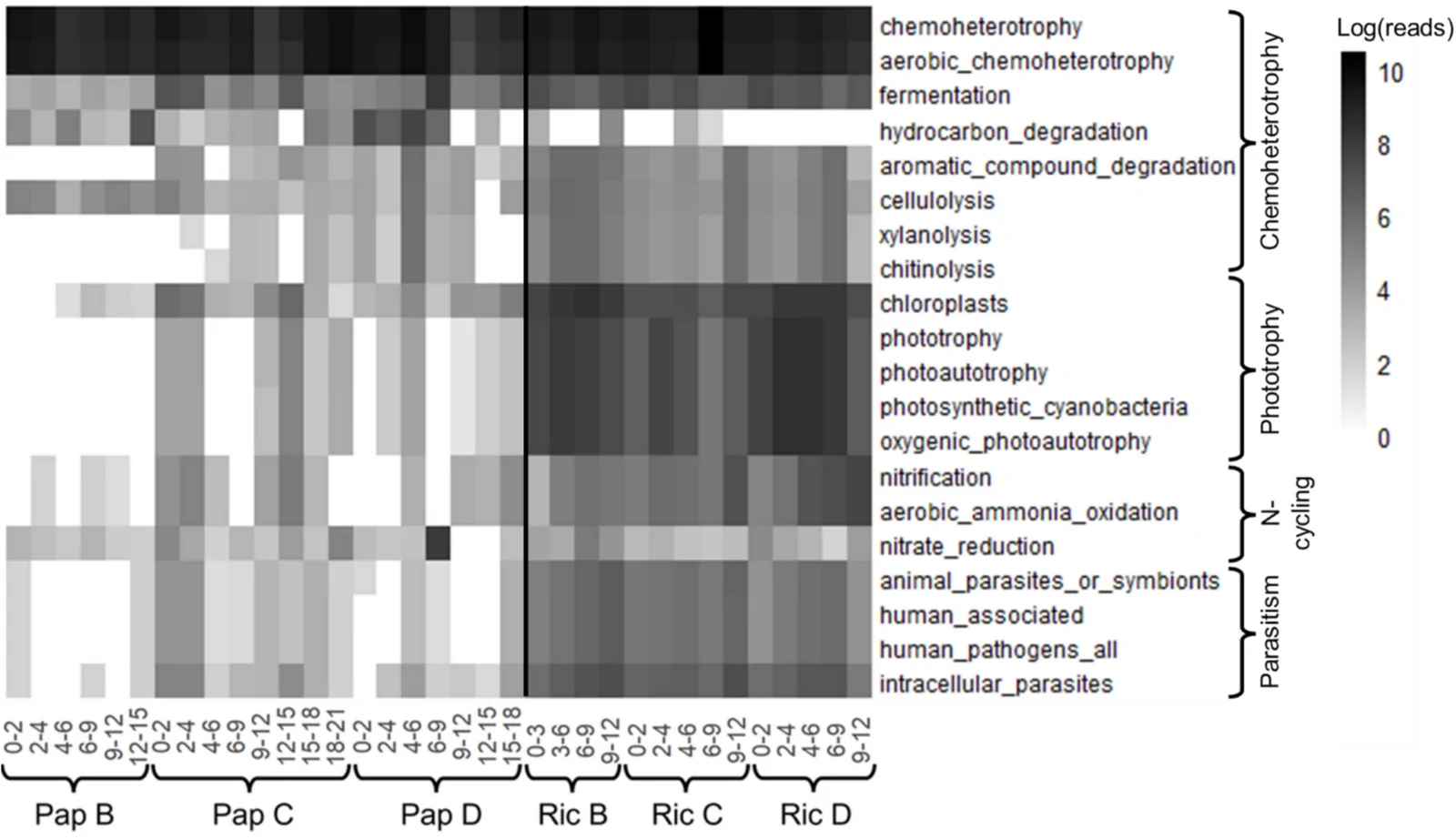
Heatmap displaying the log-transformed read counts of the top 20 putative metabolic functions (> 3000 total reads) identified via FAPROTAX. The black line separates Papakōlea (Pap) and Richardson (Ric). Sample labels indicate site (Pap or Ric), station (B, C, or D) and sediment depth in cm (e.g., 0–2, 2–4)

Using FAPROTAX, putative metabolic functions were assigned to 4564 ASVs (23% of total ASVs) represented by 969,392 reads (49% of total reads). Chemoheterotrophy was the most prominent function identified across all samples (39% of all reads assigned to a function) with the majority of these reads identified as aerobic (Fig. 6). Fermentation was assigned in all samples, representing 2% of the reads assigned. Functions related to phototrophy (including eukaryote chloroplast sequences) represented 0.8% of the total read count for Papakōlea samples, whereas their contribution to the total Richardson read count was more than 30 times higher (28%). Putative metabolic potential for nitrification also differed between the two bays and was consistently low at Papakōlea (up to 0.3% of the total assigned read count) and 9 times higher at Richardson (2.7% of the total read count).

Four samples showed a strongly deviating microbial community structure compared to other samples from the same bay. At Pap C, three sediment depths (0–2, 2–4, and 12–15 cm) displayed a community that was more diverse and differed from other Papakōlea samples (Fig. 5a, b). In one Ric C sediment layer (6–9 cm), *Psychrobacter* represented a relative abundance of 46%, while this genus was below 2% in all other samples (Fig. 5b). While the appearance of *Psychrobacter* in sample Ric C (6–9 cm) with such unusually high relative abundance is peculiar, this genus does occur in Pacific Ocean sediments [32]. Given the unmeasurable DNA concentrations in the extraction blanks (Table S1) and no visual bands for PCR blanks after gel-electrophoresis, we state that contamination of samples is an unlikely source for these deviations. In the Papakōlea NMDS, the three deviating samples are separated from others along the NMDS1 axis (Fig. 4c). However, no environmental variable vectors align clearly with this axis and thus none of our measured variables provide an explanation for this deviation. It should be noted that these three samples influence the orientation of environmental vectors in the combined NMDS ordination (Fig. 4b), particularly contributing to their upward alignment.

## Discussion

The sediment mineralogy of the two bays investigated differed from our initial expectations. Papakōlea was expected to have a high olivine content (up to 70 wt.%) due to its location within a collapsed cinder cone [22] and it being a well-known “green sand” beach. In contrast, Richardson was expected to have a higher contribution of carbonates because of the adjacent coral reef [2]. However, apart from the olivine-rich station Pap B (64 wt.%; Fig. 2c) and the carbonate-rich station Ric C (75 wt.%; Fig. 2c), the stations featured a mixture of carbonate, and volcanic-derived basalt and olivine, likely caused by along-shore sediment transport and mixing. Still, we found clear differences in the microbial community structure between the two bays, however these differences could not be directly related to the sediment mineralogy (Fig. 4b). Overall, sediment mineralogy does not appear to have a strong effect on the observed differences in microbial community structure between the two bays nor within the bay of Papakōlea (Fig. 4c). However, in the bay of Richardson, a moderate effect of mineralogy is apparent that separates the carbonate-rich station Ric C from the more olivine- and basalt-rich stations (Fig. 4d). These variations in community structure between the stations of Richardson could be driven by differences in affinity for bacterial colonization of different mineral substrates [18]. Additionally, the increased large-grain fraction (> 1 mm) at station Ric C (Fig. 2b) could lead to differences in sediment grain topography and therefore cause variation in the available microbial niches [9]. While mineralogy may influence microbial communities at finer spatial scale, broader patterns in community structure appear to be shaped more by other environmental factors.

Rather than mineralogy, the specific geomorphology and the associated differences in hydrodynamic disturbance could be responsible for the observed differences in microbial community structure. Papakōlea is completely exposed to the open Pacific Ocean, and the waves entering the bay cause bedload transport that was observed during the diving operations in our field campaign. Additionally, our campaign took place after a tropical storm period, having caused strong oceanic swell from the South. In contrast, Richardson is sheltered by lava rock beds and a coral reef (Fig. 1b) and the wave energy acting on the seabed there is much lower compared to Papakōlea. High shear stress resulting from strong hydrodynamic forces, like in Papakōlea, can prevent biofilm formation or cause detachment of existing biofilms [33, 34], thus counteracting benthic phototrophic ecosystems. In contrast, lower bed shear stress, as in Richardson, favors the stabilization of microbial communities and biofilm formation, which typically harbor a complex consortium of aerobes and photoautotrophs [33]. During field sampling, a brown cover was observed on the sediment surface in Richardson, suggesting the presence of phototrophic biofilms. This observation is supported by higher chlorophyll-*a* content, which showed the highest correlation with microbial community structure in the combined NMDS ordination (Figs. 2c and 4b). Further support is granted by the abundance of Cyanobacteria and phototrophic putative metabolic functional groups, and the presence of eukaryotic chloroplasts from diverse taxa found in Richardson (Figs. 5a and 6, Table S5). Additionally, Planctomycetes were a prominent group at Richardson; they are often found in marine biofilms and less disturbed reef sediments [33, 35, 36]. Lastly, the observed bedload transport at Papakōlea potentially lead to increased shear stress and therefore abrasion of sediment grains, whereas in the sheltered bay of Richardson, the observed sediment was of a coarser nature (Fig. 1c; Fig. S2). Cracks and dents in sediment grains serve as attachment sites and habitats that can increase microbial community diversity [9]. The higher Shannon diversity at Richardson (Fig. 4a) could therefore result from an elevated availability of microniches in the sediment.

The intense wave action at Papakōlea, as well as the coarse sediment at Richardson, is strongly associated with a high degree of advective porewater flushing, resulting in deep oxygen penetration into the sediment [11, 12]. Additionally, sampling always occurred in the troughs of sediment ripples, where oxygen concentrations are generally higher due to intrusion of oxygenated water [15]. Consequently, oxygen profiles in both bays showed oxygen saturation levels that remained > 50% throughout the top 20–25 cm of the sediment (Fig. 3). This deep oxygen availability can support the putative aerobic chemoheterotrophs that we identified in both bays, at all sediment depths (Fig. 6). Active primary production within the top layer by phototrophs may explain why oxygen saturation remained higher throughout the sediment at Richardson (Fig. 3) [11, 12], while these phototrophs were largely absent at Papakōlea (Figs. 2a and 6). Additionally, the finer sediment at Papakōlea compared to Richardson (Fig. 2b) may also explain the observed differences in oxygen saturation (Fig. 3) [12]. While an oxic-anoxic interface in the sediment typically gives a clear change in the microbial community [13, 37], this feature was not seen in our data. At Papakōlea, the microbial community structure did not vary significantly with sediment depth (down to 20 cm; Fig. 5a, b), while at Richardson, ANOSIM analysis showed a moderate difference between the surface and deeper layer. However, the predominant phyla (Proteobacteria, Bacteroidota, Plactomycetota, Firmicutes, Actinobacteriota, and Cyanobacteria) did not show large shifts along the depth profile and are all typically encountered in oxic, marine surface sediments [14, 35]. Some anaerobic micro-niches might have still been present in the interstitial spaces between sediment particles, as fermentation was also part of the predicted putative metabolic functional potential in both bays (Fig. 6).

At Richardson, ammonium was depleted in the porewater (Fig. 3), despite significant algal biomass input compared to Papakōlea (Fig. 2a). In aerobic sediments, ammonium released during organic matter degradation is rapidly nitrified [11, 38]. In Richardson, 2% of the sequence reads were associated with nitrification, compared to only 0.2% at Papakōlea (Fig. 6), suggesting a higher nitrification potential at Richardson. However, this potential was not reflected in the porewater NO_x_^−^ concentrations, which were largely comparable between bays (Fig. 3). This discrepancy may be due to the higher presence of phototrophs at Richardson, as benthic primary producers are known to take up both ammonium and nitrite/nitrate [11, 38]. Coupled nitrification–denitrification is generally suppressed in settings with benthic microalgae, due to substrate competition and increased oxygen levels, which reduces nitrogen removal through denitrification [39]. This is supported by the high oxygen levels and low potential for nitrate reduction (Figs. 3 and 6), making denitrification an unlikely sink for NO_x_^−^ at Richardson. Additionally, benthic primary producers rapidly take up phosphate [40], which could explain its depletion in the porewater at Richardson, in addition to physical porewater flushing of the coarse sediment [11]. Although we did not observe high olivine concentrations in 2/3 of the selected stations at Papakōlea (Fig. 2c), the olivine content is known to reach up to 70 wt.% in some areas of the bay [22]. Given the strong hydrodynamic transport within this bay, the higher silica concentrations observed at Papakōlea could be a consequence of olivine dissolution occurring [41]. Furthermore, diatoms were present at Richardson (Table S5) and use dissolved silica as a key nutrient, incorporating it in their cell walls [42]. Thus, benthic primary production at Richardson likely also contributed to the observed differences in silica concentrations (Fig. 3). In summary, the interplay between organic matter input, nitrification and benthic primary production at Richardson likely explains the observed differences in porewater nutrient concentrations compared to Papakōlea.

Interestingly, the dominant genus present in all Papakōlea samples was *Bacillus* (Fig. 5b), which has been associated with enhanced dissolution of olivine in terrestrial ecosystems [43, 44]. Certain *Bacillus* species can release organic ligands (e.g., siderophores) that target the iron or magnesium ions incorporated in the silicate mineral structure, thereby drastically enhancing weathering rates [7, 44, 45]. A link between microbial activity and weathering of silicate minerals is documented in terrestrial ecosystems [46]. However, so far, evidence for such a link in coastal systems is lacking [47], as no specific research efforts have been devoted to this subject. Future microbial field studies providing higher taxonomic resolution could focus on whether the *Bacillus* species found at Papakōlea are related to those involved in terrestrial silicate solubilization.

To conclude, the microbial communities differed significantly between Papakōlea and Richardson with regards to taxonomy, and to a lesser degree, in terms of their putative metabolic functional potential. While the putative metabolic functional potential assigned by FAPROTAX provides valuable insights, misannotation of functions can occur, and assignments are based on the assumption that uncultured strains share identical metabolic traits with their cultured counterparts in the database [48]. Additionally, only ~ 50% of reads in our dataset were assigned to a putative metabolic function. Nevertheless, the correspondence between FAPROTAX, taxonomic and geochemical data revealed meaningful patterns in our study. The main difference in community structure between the two bays lies in the higher prevalence of phototrophic organisms at Richardson, which we ascribe to a substantially lower degree of hydrodynamic disturbance and bed shear stress. The variations in community structure between the two bays are thus likely driven by differences in bay morphology and orientation relative to prevailing wind and wave conditions. The consequent differences in sediment disturbance then generate sorting effects and differences in grain size distribution, ultimately providing variation in microbial niches. While mineralogy did correlate to microbial community structure in Richardson, no such link was apparent between the two bays, suggesting that mineralogy can play a role in shaping microbial communities at finer spatial scale, but that its impact is of lesser importance when comparing different geographic locations, in which environmental factors such as wave exposure, organic matter input, and grain size are of greater importance for shaping microbial community structure [17]. Lastly, our results highlight the complexity of marine sediment environments, which poses a significant challenge for monitoring, reporting, and verification (MRV) of field studies on olivine-based ocean alkalinity enhancement. The inherent heterogeneity of marine sediment environments complicates the identification of suitable study and control sites for field trials, emphasizing the need for careful site selection.

## Supplementary Information

Below is the link to the electronic supplementary material.

Supplementary table S1 (CSV 7 KB)

Supplementary table S2 (CSV 1 KB)

Supplementary table S3 (CSV 8 KB)

Supplementary table S4 (CSV 1 KB)

Supplementary table S5 (CSV 7 KB)

Supplementary information document (DOCX 6619 KB)

## Data Availability

All sample metadata, mineralogy, grain size, porewater, Shannon diversity, envfit correlations, and BLASTn data are available in the supplementary tables (Online Resource). Descriptions of these supplementary tables are presented in the Supplementary Information, alongside Figure S1, S2, S3 and the supplementary methods. Sequencing data were uploaded to the NCBI SRA Bioproject PRJNA1217334.
