# Supplementary material for: Microbial Community Structure in Contrasting Hawaiian Coastal Sediments
Source: Microb Ecol. 2025 May 23;88(1):51. doi: 10.1007/s00248-025-02548-7 (PMC12102127; doi:10.1007/s00248-025-02548-7)
Supplement: Supplementary file 6 — Supplementary information document (DOCX 6619 KB) [file 248_2025_2548_MOESM6_ESM.docx]

**Supplementary information**

**­Title**: Microbial community structure in contrasting Hawaiian coastal sediments

**Authors**: Benjamin Van Heurck^1^, Diana Vasquez Cardenas^1^, Astrid Hylén^1^, Emilia Jankowska^2,3^, Devon B. Cole^2,3^, Francesc Montserrat^2^, Matthias Kreuzburg^1,4^, Stephen J. Romaniello^2^, Filip J. R. Meysman^1^

**Corresponding author:** BVH – [benjamin.vanheurck@uantwerpen.be](mailto:benjamin.vanheurck@uantwerpen.be) – ORCID 0000-0001-6199-1627

**Affiliations**:

1. Geobiology research group, department of Biology, University of Antwerp,
Universiteitsplein 1, 2610 Antwerp, Belgium

2. Vesta, PBC, San Francisco, California, USA

3. Current affiliation - Hourglass Climate, NPO, Montclair, New Jersey, USA

4. Current affiliation - Department of Marine Chemistry, Trace Gas Biogeochemistry, Leibniz-Institute for Baltic Sea Research, Warnemünde, Germany

5. Current affiliation – ARK Rewilding Nederland, Winselingseweg 95, 6541 AH Nijmegen, The Netherlands

**Journal:** Microbial Ecology

**Description:**

This document contains supplementary figure S1, displaying the method of porewater collection, figure S2 showing sediment from all sampled stations, and figure S3 of the rarefaction curves for the sequence data, as well as all supplementary methods with detailed information on sample processing, Shannon diversity calculation and BLAST results. All supplementary tables are available as an Online Resource. Supplementary table S1 contains information on microbial samples, processing and sequencing data analysis, Shannon Diversity and microbial metadata, table S2 displays the grain size and mineralogy data, table S3 contains the porewater data, table S4 indicates the correlation coefficients and p-values for the vectors fitted over the NMDS ordination of Pap+Ric, Pap and Ric, and table S5 shows the results of the BLASTn search.

**Supplementary figures:**

**
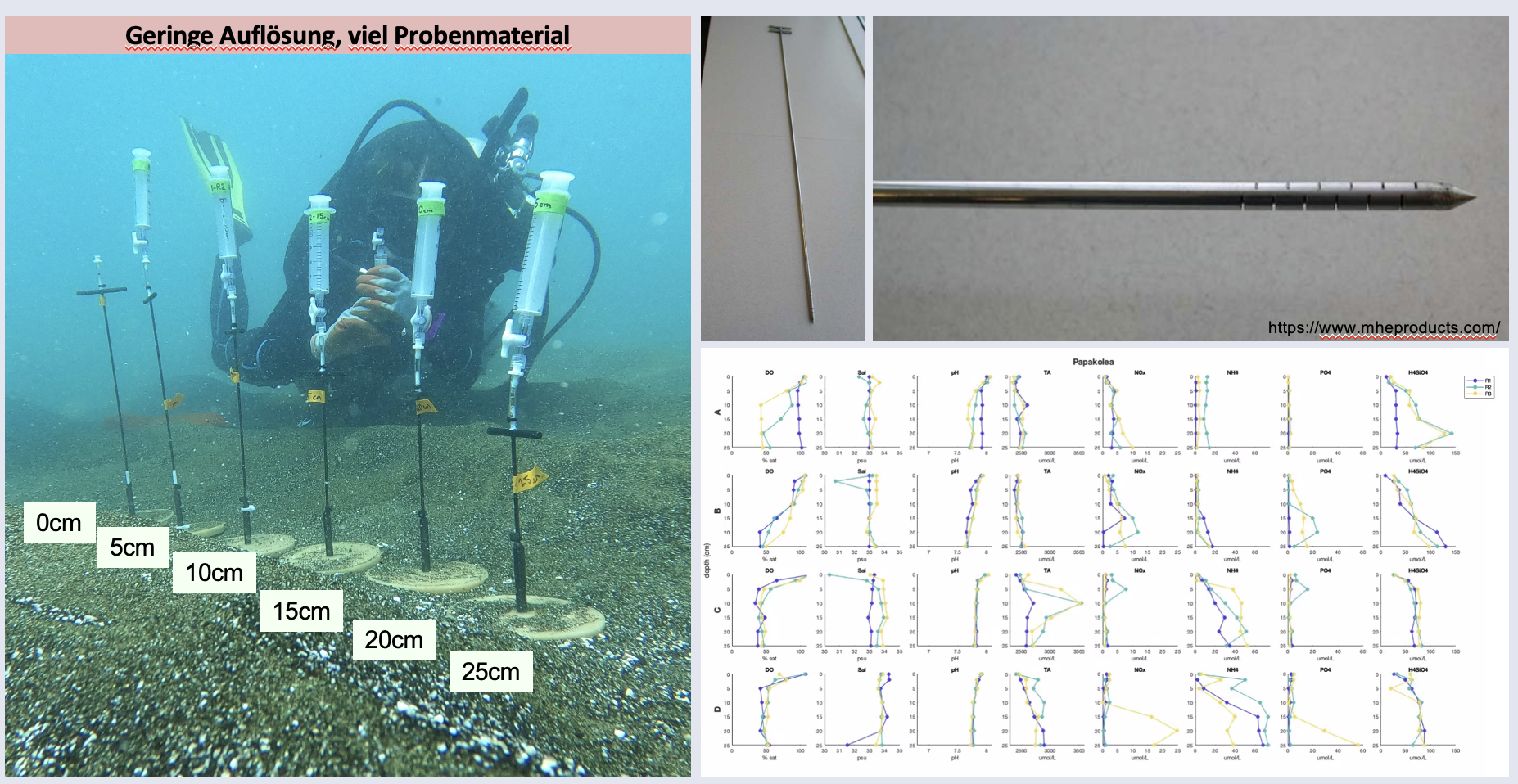
**

**Figure S1** Picture showing the method for porewater sampling by SCUBA divers. Water was collected into syringes via carbon fiber sippers inserted at 2, 5, 10, 15, 20 and 25 cm depth ([https://www.mheproducts.com/](https://eur01.safelinks.protection.outlook.com/?url=https%3A%2F%2Fwww.mheproducts.com%2F&data=05%7C02%7CBenjamin.VanHeurck%40uantwerpen.be%7C81ea31e824684fa6c1af08dd6175df99%7C792e08fb2d544a8eaf72202548136ef6%7C0%7C0%7C638773881188250236%7CUnknown%7CTWFpbGZsb3d8eyJFbXB0eU1hcGkiOnRydWUsIlYiOiIwLjAuMDAwMCIsIlAiOiJXaW4zMiIsIkFOIjoiTWFpbCIsIldUIjoyfQ%3D%3D%7C0%7C%7C%7C&sdata=zwS7%2FBfuu7JAIAc20T%2FAE32MT4imhNjlKU%2FP7CiLhuQ%3D&reserved=0)). Bottom water is represented by 0 cm depth. The row of carbon fiber sippers was moved 1 m in between replicate porewater profiles. Photograph taken and edited by Matthias Kreuzburg


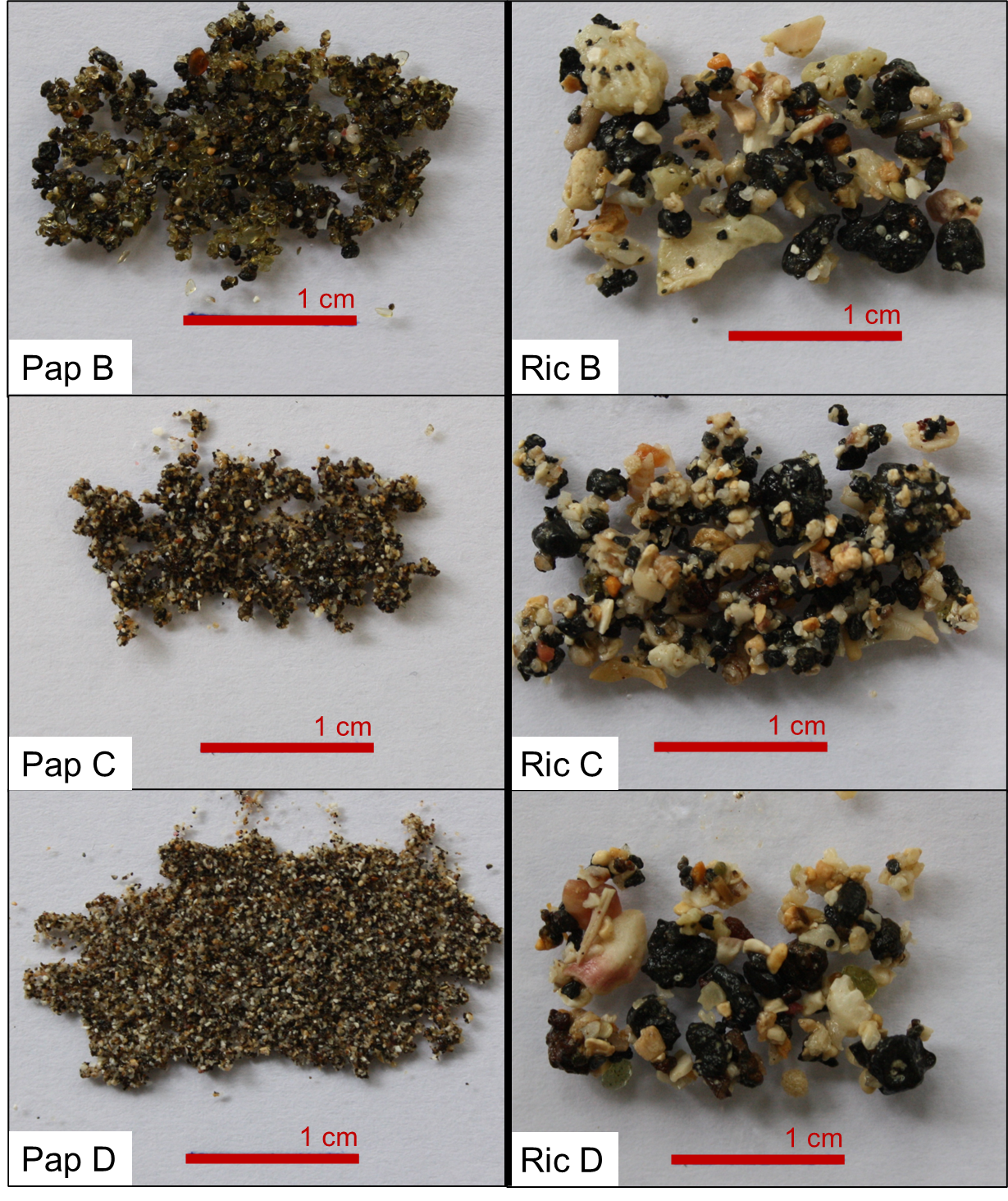


**Figure S2** Pictures showing sediment of Papakōlea (left) and Richardson (right) stations B, C and D. Sediment was collected from the 4-6 cm layer


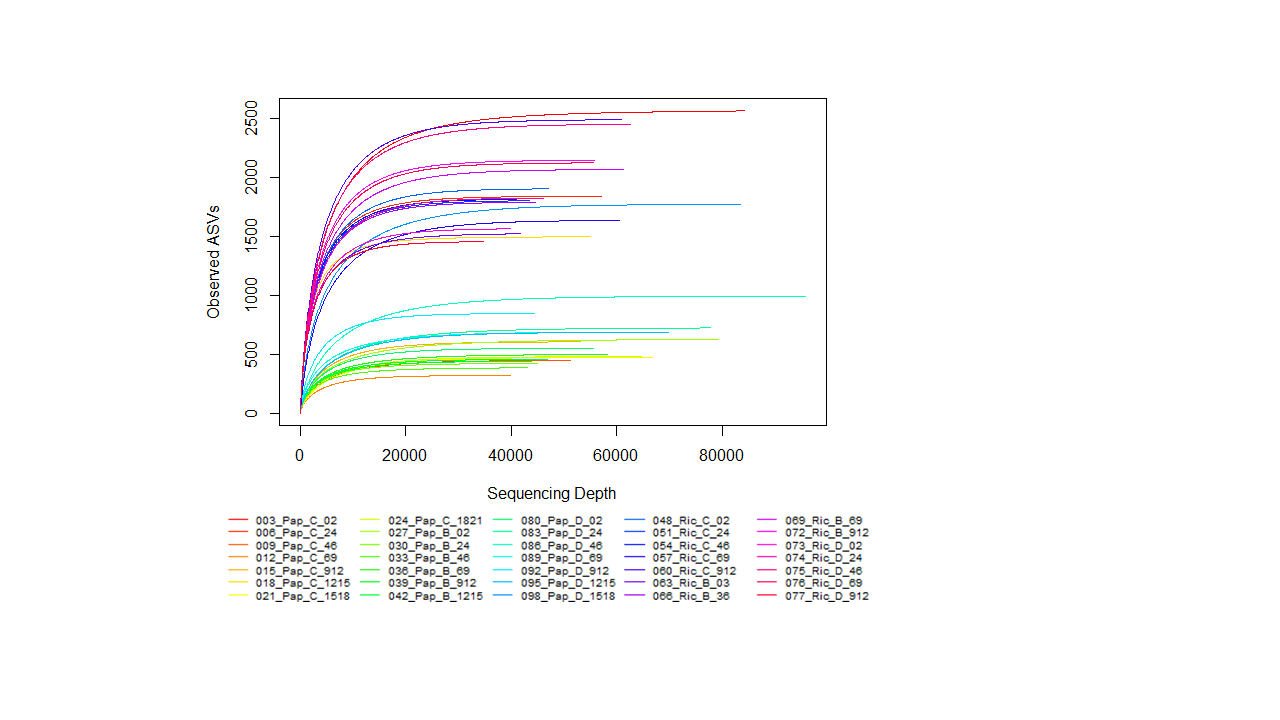


**Figure S3** Rarefaction curve showing sequencing depth (x-axis) vs. number of ASVs (y-axis) for Papakōlea and Richardson samples. After rarefaction, equal sampling depth for all samples was obtained at 34699 reads. Rarefied samples were used to calculate Shannon diversity.

**Supplementary methods:**

DNA extraction:

Following adaptations were made to the standard extraction protocol from Qiagen: a) 10-minute incubation step at 65 °C was added prior to the vortex step, b) the vortex step was executed for ~15 minutes, c) elution volume of 30 µl was used. DNA concentrations of samples were measured using an Invitrogen™ Qubit™ 3.0 Fluorometer (Life Technologies, Carlsbad, CA, USA) and ranged from 0.2 – 2.6 ng/µL (Table S1). One sample from Pap B (sediment layer: 15-18 cm) was excluded from further analysis as no measurable DNA concentration was obtained after multiple extraction attempts. Extraction blanks yielded no measurable DNA concentration.

PCR:

Following cycling parameters were used in the PCR: initial denaturation 1 min at 98 °C, cyclic denaturation 30 sec at 98 °C, annealing 30 sec at 53 °C, cyclic extension 30 sec at 72 °C and final extension 5 min at 72 °C, with 30 or 32 cycles (Table S1). Gel-electrophoresis on a 1.5% agarose gel (5 min at 70V and 35 min at 100V) and subsequent staining with SYBR™ Gold Nucleid Acid Gel Stain (Thermo Fischer Scientific, Waltham, MA, USA) were used for quality control of PCR products. PCR products were then enzymatically cleaned with ExoCleanUp FAST (VWR International, Radnor, PA, USA) at a ratio of 5:1 (reaction conditions: 15 min at 37 °C and terminated for 10 min at 80 °C). DNA concentrations were measured using an Invitrogen™ Qubit™ 3.0 Fluorometer. For samples that failed to reach a >2 ng/µl threshold, PCR volume was increased (20 µl or 25 µl instead of 10 µl) and the termination step at 80 °C was reduced to 5 minutes (Table S1).

Microbial community analysis:

Primers and adapters were removed from sequencing reads using the ‘trimLeft’ argument in the dada2 ‘filterAndTrim’ function. Prior to the DADA2 algorithm, forward and reverse reads were truncated at 265 and 175 respectively. After running the DADA2 algorithm, reads were merged and chimeras were removed, thereby retaining an average of 72 ± 8% of the input sequences (Table S1). Singleton sequences were removed prior to further analysis. A rarefaction curve was constructed (Fig. S3) and data were rarefied to the minimum sequencing depth (34,699 reads) prior to calculating Shannon diversity. This resulted in loss of 514 ASVs (2.5% of total ASVs). For NMDS and construction of bar plots, read count data were not rarefied, but transformed to relative abundances. These relative ASV abundances were used to identify the top 20 most abundant taxa at phylum and genus level. After obtaining a putative metabolic functional table using FAPROTAX, functions with a total assigned number of sequences lower than 3000 across all samples (<0.3% of total reads across all samples) were excluded from further analysis. Read counts per putative metabolic function were log-transformed and visualized with a heatmap.
